# Pleiotropic Effects of Caffeine Leading to Chromosome Instability and Cytotoxicity in Eukaryotic Microorganisms

**DOI:** 10.4014/jmb.2011.11042

**Published:** 2020-12-25

**Authors:** Woo-Hyun Chung

**Affiliations:** 1 College of Pharmacy, Duksung Women’s University, Seoul 0369, Republic of Korea; 2 Innovative Drug Center, Duksung Women’s University, Seoul 01369, Republic of Korea

**Keywords:** Caffeine, growth inhibition, radiosensitization, DNA damage checkpoint, unicellular eukaryotes

## Abstract

Caffeine, a methylxanthine analog of purine bases, is a compound that is largely consumed in beverages and medications for psychoactive and diuretic effects and plays many beneficial roles in neuronal stimulation and enhancement of anti-tumor immune responses by blocking adenosine receptors in higher organisms. In single-cell eukaryotes, however, caffeine somehow impairs cellular fitness by compromising cell wall integrity, inhibiting target of rapamycin (TOR) signaling and growth, and overriding cell cycle arrest caused by DNA damage. Among its multiple inhibitory targets, caffeine specifically interacts with phosphatidylinositol 3-kinase (PI3K)-related kinases causing radiosensitization and cytotoxicity via specialized intermediate molecules. Caffeine potentiates the lethality of cells in conjunction with several other stressors such as oxidants, irradiation, and various toxic compounds through largely unknown mechanisms. In this review, recent findings on caffeine effects and cellular detoxification schemes are highlighted and discussed with an emphasis on the inhibitory interactions between caffeine and its multiple targets in eukaryotic microorganisms such as budding and fission yeasts.

## Introduction

Caffeine (1,3,7-trimethylpurine-2,6-dione) is a naturally occurring purine derivative found in beverages such as coffee, tea, energy drinks, and several medications, as well as a myriad of dietary sources [1]. In higher animals, caffeine acts as a non-selective antagonist for A_1_ and A_2A_ adenosine receptors in both heart and brain and has antidepressant and diuretic effects [2, 3]. Low doses of caffeine (< 65 mg) have been known to improve cognitive performance, working memory, and mood, whereas approximately 200 mg increases alertness, arousal, task accuracy, and energy in normal human populations [4, 5].

Caffeine is known to affect cell growth, proliferation, and energy metabolism by inhibiting the mammalian target of rapamycin (mTOR) signaling pathway [6]. Caffeine elicits pleiotropic physiological responses by triggering intracellular Ca^2+^ mobilization in various cell types [7]. Caffeine also inhibits DNA damage repair and perturbs cell cycle checkpoint function, which might lead to mutagenesis, apoptosis, and carcinogenesis [8]. Many earlier studies indicated that caffeine impairs cell cycle progression delays induced by chemicals or radiation, and enhances the toxicity of anti-cancer agents [9, 10]. Controversially, however, an accumulating body of evidence suggests that caffeine may both enhance and antagonize carcinogenic effects [11].

Caffeine elicits chemopreventive effects in mouse skin tumorigenesis models by inducing apoptosis [12]. Caffeine specifically suppresses epidermal growth factor (EGF)-induced malignant cell transformation and promotes human keratinocyte apoptosis with unrepaired DNA damage by blocking ultraviolet B (UVB)-induced phosphorylation of Chk1 and AKT, thereby preventing tumorigenesis. However, the mechanisms by which these effects are triggered remain largely unknown [13, 14]. Recently, it has been reported that caffeine enhances anti-tumor immune responses in mice by blocking the A_2A_ receptor [15].

Several meta-analyses have reported that there is no significant association between caffeine intake and ovarian cancer risk [16]. Nonetheless, caffeine consumption is somehow inversely associated with the incidence of melanoma, liver and endometrial cancer, and type 2 diabetes in a dose-dependent manner [17-19]. Therefore, despite extensive analyses, the effects of caffeine on cell cycle progression and proliferation remain ambiguous.

Caffeine has also been reported to inhibit bacterial growth. Particularly, earlier studies demonstrated that caffeine impairs thymidine metabolism, thereby inhibiting DNA synthesis in *Escherichia coli* [20, 21]. At high doses, caffeine effectively kills *E. coli* and *Salmonella enterica*, albeit not as effectively as standard antibiotic ampicillin, and is more effective against *Staphylococcus aureus* and *Enterobacter aerogenes* [22-24]. Caffeine is a secondary metabolite produced by over one hundred plant species and possesses antibacterial activity against several plant pathogenic bacteria such as *Ralstonia solanacearum*, *Clavibacter michiganensis*, *Xanthomonas campestris*, and others. [25]. Caffeine alters cell morphology, substantially delays cell division time, and suppresses RNA biosynthesis [25]. Intriguingly, however, *Pseudomonas aeruginosa* and *Pseudomonas putida* can reportedly degrade caffeine to use it as a nutrient and carbon source [26].

Yeast, a relatively simple unicellular eukaryote, is also responsive to caffeine. Specifically, caffeine affects yeast cell growth, morphology, and a variety of cellular metabolic pathways to maintain homeostasis [27]. High concentrations of caffeine act as a cell wall antagonist triggering the mitogen-activated protein (MAP) kinase cascade for cell wall integrity signaling, and exert mutagenic effects leading to cell cycle alterations through the suppression of Tel1 and Mec1, two yeast homologs of mammalian ataxia-telangiectasia mutated (ATM) and ATM-related (ATR) kinases [28, 29].

Despite its use as a genotoxic agent over 40 years, the molecular mechanisms underlying the adverse effects of caffeine on cell proliferation and maintenance have not been elucidated. This review on the pleiotropic effects of caffeine on unicellular eukaryotes provides a comprehensive overview of how cells respond to stressful environmental conditions and toxic substances similar to caffeine via complex cellular processes, including cell signaling, cell cycle regulation, and damage checkpoint activation.

## Caffeine Has Multiple Targets that Impair Diverse Cellular Mechanisms

**Fig. 1:**
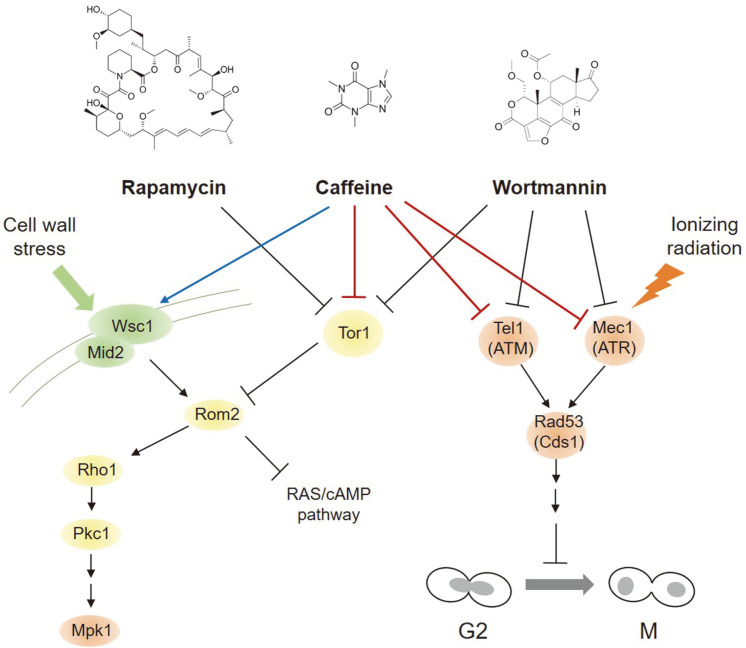
Schematic diagram showing how caffeine and its related drugs affect Pkc1-Mpk1 kinase pathway, Tor1-mediated signaling, and Tel1/Mec1 (ATM/ATR in mammals)-mediated damage checkpoint responses by inhibiting PI3K-related protein kinases in *S. cerevisiae* cells. Proteins mainly localized to cell wall and outer membrane are colored in green; cytoplasm in yellow; and nucleus in orange. G2; cell cycle phase Gap 2, M; mitosis.

Caffeine is a relatively non-selective agent and affects multiple cellular processes related to cell growth and metabolism, mostly by acting as a low-affinity adenosine analog [30]. The physiological activity of caffeine has been reported to inhibit alkaline phosphatase, cAMP phosphodiesterase, and the nucleotide exchange activity of RCC1 (regulator of chromosome condensation) [31-34]. In yeasts and fungi, caffeine is categorized as a cell wall perturbing agent, such as Congo red and Calcofluor-white, as mutants that lack cell surface sensors or components of the MAP kinase cascade of the cell wall integrity pathway are sensitive to caffeine [35, 36]. Caffeine induces rapid phosphorylation of Mpk1, the downstream MAP kinase of the Pkc1-mediated cell wall integrity pathway in yeast, and Mpk1 phosphorylation by caffeine is abolished in Tor1 kinase-defective mutant cells (Fig. 1). Genetic and biochemical data from genome-wide transcriptome analyses have shown that caffeine activates a subset of cell wall-related genes through the Pkc1-Mpk1 cascade and inhibits the Ras/cAMP pathway through Tor1-mediated signaling [37, 38].

TOR signaling is a well-known pathway for cellular homeostasis and growth, and caffeine exhibits a remarkably similar effect to that of rapamycin on the inhibition of TOR complex 1 (TORC1) and the ensuing alteration of global gene expression patterns in yeast [39]. Mutant cells lacking the genes encoding Tor1, Kog1, or Tco89, three non-essential TORC1-specific components, exhibit hypersensitivity to caffeine, suggesting that TORC1 is a specific caffeine target [39, 40]. Caffeine, wortmannin, and many other compounds with similar structures markedly inhibit the phosphorylation of mammalian TOR (mTOR)-dependent substrates both in vivo and in vitro [41]. Wanke *et al*. [42] proposed that caffeine extends the life span of yeast cells by targeting TORC1 and its downstream kinase cascade. TOR kinase belongs to the phosphatidylinositol 3-kinase-related kinase (PIKK) family, which commonly contains a c-terminus serine/threonine protein kinase domain similar to the catalytic domain of phosphatidylinositol 3-kinases (PI3Ks) [43]. This family includes ATM, ATR, and DNA-dependent protein kinase (DNA-PK), the catalytic activity of which can be inhibited to varying degrees by various xanthine alkaloids, including caffeine [44, 45].

Caffeine inhibits sugar transport by binding at the nucleotide-binding site of GLUT1, the primary facilitative glucose transporter in mammals [46]. GLUT1 is also allosterically inhibited by ATP, and AMP acts as a competitive antagonist of ATP-mediated glucose uptake inhibition. Interestingly, kinetic analyses have revealed that ATP can antagonize caffeine-mediated uncompetitive inhibition of glucose uptake, suggesting that caffeine and adenosine share structural similarities [47].

Caffeine also regulates calcium mobilization by inhibiting extracellular Ca^2+^ uptake in *S. cerevisiae* [48]. Caffeine directly binds to and inhibits voltage-gated Ca^2+^ channels, and may have indirect effects by inhibiting cAMP phosphodiesterase, resulting in PKA-dependent inhibition of Ca^2+^ channels [48, 49]. Lack of components in the calcineurin pathway also led to caffeine sensitivity in fission yeast, further suggesting that Ca^2+^ levels are regulated by caffeine [50].

Fcy2, a purine-cytosine permease, is thought to act as a non-specific transporter for caffeine uptake in *S. cerevisiae*, as intracellular caffeine accumulation is not observed in *fcy2* mutant cells even when treated with high doses [51]. Due to its structural similarity to adenine, guanine, hypoxanthine, and cytosine, all of which are readily uptaken by cells, caffeine could affect several nucleic acid metabolic pathways including both DNA synthesis and degradation mechanisms involved in DNA replication and damage repair. In fact, caffeine reportedly alters cell cycle control by inducing mitosis even before DNA replication is completed in mammalian cells. Moreover, caffeine also potentiates the lethal effects of several genotoxic agents primarily due to inhibition of timely DNA repair [52]. For instance, synchronized BHK and CHO hamster fibroblasts arrested in the early S phase with hydroxyurea (HU) underwent premature mitotic events when exposed to caffeine [53, 54]. Additionally, caffeine was shown to override S-M checkpoint induction by inhibiting Cds1 activation and Chk1 phosphorylation in the fission yeast *Schizosaccharomyces pombe* [55].

## Caffeine Acts as a DNA Damage-Sensitizing Agent

It has been reported in earlier studies that caffeine acts as a DNA damage repair inhibitor and reduces the duration of radiation-induced cell cycle arrest in the G2 phase [10, 56]. Although caffeine alone did not affect timely cell cycle progression, caffeine led to radiosensitization to X-rays and induced G2/M override in several mammalian cell lines, which was more pronounced in p53 null cells than their wild-type counterparts [57-59]. As p53-deficient cells fail to arrest at G1, irradiation-induced cell cycle control becomes completely dependent on G2 arrest, which is abrogated by caffeine-mediated activation of Cdk1 (also known as Cdc2), leading to sensitization to apoptosis [60]. Given that p53-deficient primary and tumor cells can be preferentially vulnerable to DNA damage-inducing reagents with caffeine exposure, caffeine could serve as a useful anticancer genotoxic adjunct in radiation therapy and chemotherapy [61].

Caffeine is believed to have multiple molecular targets and may possess an especially high affinity to protein kinases due to its chemical properties [58]. Predictably, caffeine treatment before irradiation, but not after irradiation, inhibited the radiation-mediated activation of Cds1 and its upstream kinase ATM in HeLa cells [62]. In vitro kinase assays also revealed that caffeine directly inhibits ATM kinase, not Cds1, which was consistent with the effects of wortmannin, a selective phosphoinositide 3-kinase (PI3K) inhibitor [62, 63]. In turn, inhibition of Cds1 phosphorylation by caffeine prevents the inactivation of Cdc25, leading to activation of Cdk1 and premature G2/M transition (Fig. 1).

This DNA damage-sensitizing effect of caffeine is associated with the inhibition of multiple components of the damage checkpoint signaling machinery. The activity of another major PI3K-related kinase, ATR, is also suppressed by caffeine in vivo as well as in vitro even in the absence of its substrate DNA molecules [53, 44, 64]. In contrast, in vitro kinase assays determined that DNA-PK and hChk1 are relatively resistant to caffeine-induced radiosensitivity [44]. Several other methylxanthine-derived drugs such as theobromine, theophylline, paraxanthine, and pentoxifylline also lead to cell radiosensitization at low concentrations [44].

## Caffeine-Mediated Inhibition of PI3K-Related Protein Kinases

**Fig. 2:**
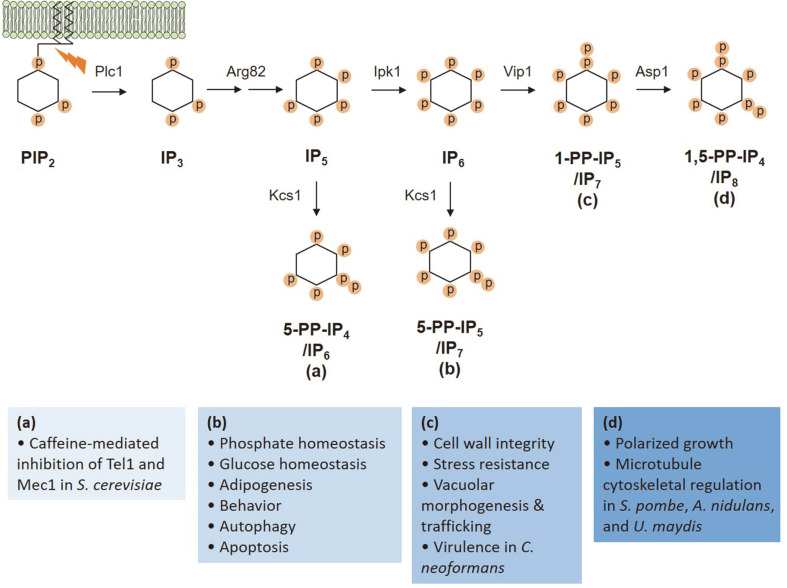
Schematic model showing major steps in inositol pyrophosphate (PP-IP) metabolism and enzymes catalyzing each converting step in *S. cerevisiae*. PP-IPs and their metabolizing enzymes regulate a variety of biological processes as shown in (a)~(d). Caffeine inhibition of Tel1 and Mec1 activity seems to be mediated by PP-IP_4_.

The lethal effects of caffeine and wortmannin via the inhibition of Mec1 and Tel1 kinase activity in *S. cerevisiae* are selectively blocked in mutants lacking inositol pyrophosphate (PP-IP) synthesis [29]. Inositol pyrophosphates are a specialized group of signaling molecules that are highly conserved from yeast to higher eukaryotes and regulate numerous energetic biological processes such as cell growth, environmental stress responses, pathogenicity, and autophagy (Fig. 2) [65-68]. The budding yeast *kcs1* mutant, which is unable to produce high energy inositol pyrophosphates, displays slow growth, defective endocytic trafficking, and resistance to caffeine and wortmannin [69]. Moreover, inositol polyphosphate multikinase (encoded by *ARG82*)- or phospholipase C (*PLC1*)-deficient mutant strains are also resistant to the harmful effects of caffeine, whereas the *ipk1* mutant, which lacks inositol polyphosphate kinase 1, is not caffeine resistant [29]. These observations suggest that the lethal effects of caffeine are mediated by a specific form of inositol pyrophosphate such as PP-IP_4_ (Fig. 2).

In order to respond to a wide variety of stimuli, the high-energy pyrophosphate group of inositol pyrophosphates might conceivably drive or inhibit phosphotransfer reactions even in a kinase-independent manner, thus affecting signaling via the PI3K-related protein kinase family [70]. Inositol pyrophosphates produced by mammalian IP6 kinase 1 impact insulin sensitivity and weight gain by inhibiting Akt, a serine/threonine-specific protein kinase [71]. The *kcs1* yeast mutant displays longer telomeres, which is mainly regulated by PI3K-related protein kinase, Tel1 [29, 72]. Altogether, these results indicate that methylxanthine-induced inhibition of Mec1 and Tel1 might be involved in the suppression of their phosphotransferase activity mediated by the specific group of inositol pyrophosphates [44]. Intriguingly, however, *Cryptococcus neoformans* mutant strains lacking Ipk1 and/or Kcs1 show a significant growth defect in the presence of caffeine, which is inconsistent with the resistant phenotypes observed in *S. cerevisiae* [73].

In contrast, Cortez [74] reported that neither ATM nor ATR activity in vivo is inhibited by caffeine. Caffeine treatment abrogated ionizing radiation (IR)- or hydroxyurea (HU)-initiated G2/M checkpoint activation without any decrease in ATM- or ATR-dependent phosphorylation of CHK1 and CHK2 in human cell lines. In line with these observations, caffeine has also been found to intercalate into DNA molecules and prevent the binding of damage repair proteins, thereby interfering with DNA repair activities and promoting hyperactivation of ATM and ATR due to feedback mechanisms [52, 75].

Although checkpoint abrogation could provide a mechanistic explanation for caffeine-induced radiosensitization, relevant studies have only identified a relatively weak correlation between checkpoint disruption and caffeine-induced radiosensitization levels [76, 77]. Moreover, ataxia-telangiectasia (A-T) cells defective in ATM are still significantly radiosensitized by caffeine treatment, suggesting that other factors might mediate caffeine-induced cytotoxicity, including the inhibition of DNA repair systems [78].

## Caffeine Inhibits DNA Damage Repair Pathways

**Fig. 3:**
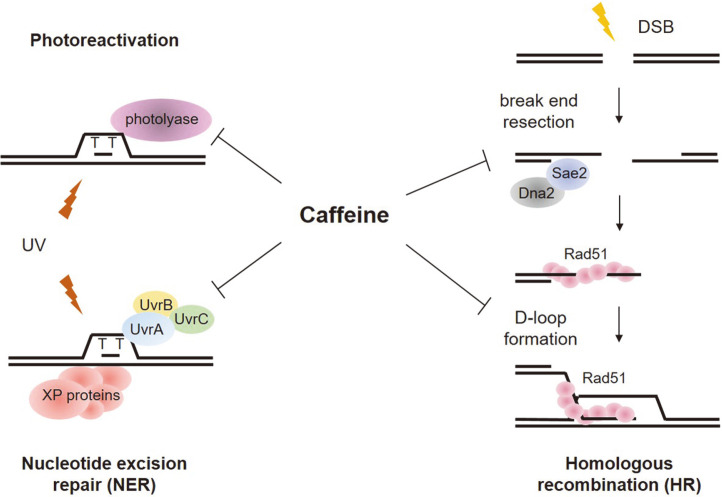
Inhibitory effects of caffeine on various DNA damage repair proteins. Caffeine inhibits repair of pyrimidine dimers interfering with the binding of DNA photolyase and UvrA to damaged DNA lesions. Caffeine also inhibits several pivotal steps in HR pathway by removing Sae2 and Dna2 nucleases, and by promoting non-productive Rad51 nucleofilament formation.

Among several DNA damage repair mechanisms, photoreactivation and nucleotide excision repair (NER) were reportedly inhibited by caffeine, whereas none of the tested major repair enzymes for base excision repair (BER) were affected [52, 79]. Caffeine inhibits photoreactivation by interfering with the binding of DNA photolyase to damaged DNA lesions (Fig. 3). Interestingly, however, NER-mediated repair is inhibited by caffeine because it promotes nonspecific binding of UvrA, the damage recognition subunit of ABC excinuclease in *E. coli*, to undamaged sites, thereby inhibiting proper nicking of damaged DNA (Fig. 3) [52]. The inhibitory effect of caffeine is also prominent in xeroderma pigmentosum (XP)-variant cells in humans, which can be reversed by the expression of DNA polymerase η, a crucial component for accurate translesion DNA synthesis through bulky DNA damage caused by UV radiation [80, 81]. Other DNA intercalating agents such as ethidium bromide and acridine orange also inhibit excinuclease activity through a similar mechanism, suggesting that caffeine inhibits DNA repair by intercalating into DNA and distorting its native helical structure [52]. This conclusion is consistent with the observation that BER enzymes, which are involved in small-scale base damage repair, are not largely affected by caffeine treatment.

The inhibitory effects of caffeine on homology-dependent damage repair have been previously documented in many organisms [82-84]. Several pivotal steps in homologous recombination (HR) for DSB repair are inhibited by caffeine. It has been observed in budding yeast and HeLa cells that caffeine impairs DSB-induced DNA end resection by rapid loss of Sae2 and Dna2, two nucleases that play important roles in early stages in the homologous recombination pathway (Fig. 3) [85]. The amount of functional Sae2 and Dna2 is reduced by caffeine-induced proteasomal degradation even in the absence of DNA damage, and this effect is independent of DNA damage checkpoint inhibition. It is also worth noting that autophagy can be stimulated in yeasts and mammalian cells by caffeine, as well as rapamycin and valproic acid (VPA), which are well-known autophagy-inducing agents [86-88].

Consistent with earlier studies on caffeine toxicity in recombination-dependent DNA repair, genome-wide screening of caffeine-sensitive mutants in the fission yeast has shown that *rad3*, *ssb3*, *rad51*, and *rad54* mutants defective in homologous recombination are all sensitive to caffeine [50]. In fact, caffeine disturbs gene targeting by promoting non-productive Rad51 nucleofilament formation with random genomic regions (Fig. 3). As a result, caffeine repels the previously assembled Rad51 foci [89]. Caffeine interferes with Rad51-mediated strand exchange during DNA repair by homologous recombination (HR), whereas γH2AX and Exo1 remain activated at DNA breaks, which renders cells more sensitive to genome instability [90, 91]. Homologous recombination interference by low caffeine concentrations (2~4 mM) appears to be independent of its inhibition of Tel1- or Mec1-mediated damage checkpoint responses, suggesting that caffeine does not affect gene targeting by specific checkpoint inhibition but rather by different mechanisms. Moreover, DNA replication inhibition, another previously reported caffeine effect, was not observed upon caffeine treatment at similar concentrations as those mentioned above [90].

**Fig. 4:**
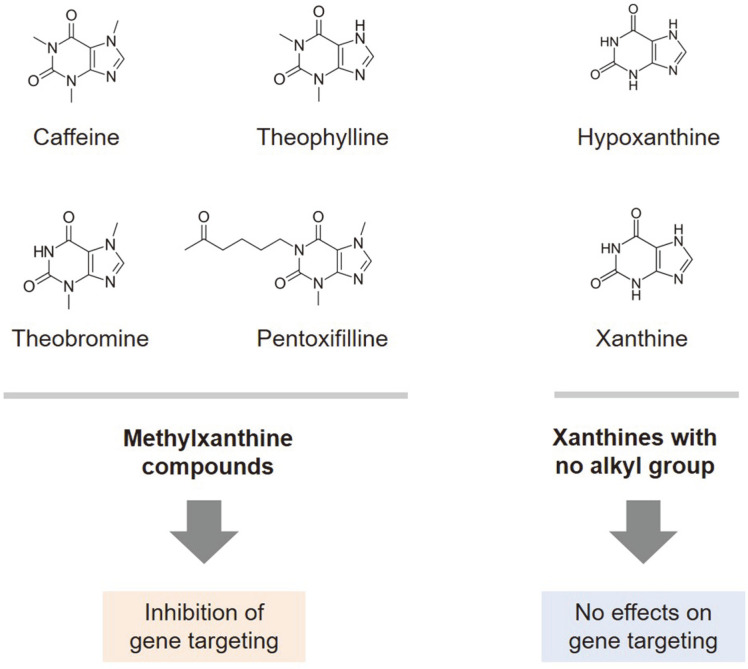
Effects of caffeine and its structurally-related compounds on gene targeting efficiency via Rad51-mediated homologous recombination. Methylxanthines, but not xanthines with no alkyl groups, suppress Rad51-mediated D-loop formation and strand exchange.

The aforementioned observations have led to speculation regarding the specificity of caffeine-mediated inhibition. Interestingly, some related methylxanthine compounds lacking only one methyl group compared to caffeine, such as theophylline, theobromine, and pentoxifylline, exhibit similar or even more severe inhibitory effects on homologous recombination than caffeine, which are consistent with the results of checkpoint activation inhibition experiments, whereas hypoxanthine and xanthine with no alkyl groups have almost no negative effects on gene targeting efficiency (Fig. 4) [90, 92]. Suppression of homologous recombination is caused by alkylxanthine-specific inhibition of D-loop formation, which has been demonstrated as not being due to intercalation-induced topological DNA structure change but to the direct formation of non-productive and homology-independent Rad51 nucleoprotein filaments [90].

Caffeine-induced radiosensitizing effects are significantly diminished in mutant cells deficient in *XRCC2* and *XRCC3*, as well as in two *RAD51* paralogs in mammals [78, 93]. Additionally, the strong inhibition of homology-directed repair by caffeine observed in wild-type cells was substantially reduced in mutant cells lacking *XRCC2* and *XRCC3*. Moreover, these mutants show nearly intact damage checkpoint responses, i.e., timely G2 arrest and delayed S phase. Intriguingly, no significant effect of caffeine has been experimentally measured on non-homologous end joining (NHEJ) [78]. Together, these observations suggest that checkpoint disruption by itself is not sufficient for radiosensitization and the inhibition of homologous recombination proteins is likely an additional component of the caffeine radiosensitization mechanisms.

## Integrated Intracellular Mechanisms Conferring Caffeine Tolerance

Screening for caffeine-resistance genes using a *S. cerevisiae* multicopy vector library revealed that two multidrug resistance (MDR) transporters, Snq2 and Pdr5, mediate caffeine efflux in yeast [94]. Snq2 is functionally homologous to Pdr5, which is one of the most abundant ATP-binding cassette (ABC) transporters involved in cellular detoxification in yeast; overexpression of either one of them is sufficient to confer resistance to caffeine although Snq2 is slightly more effective [94, 95]. Another set of studies showed that combinatorial deletions of the *HSE1*, *RTS3*, *SDS23*, and/or *SDS24* genes, none of which are involved in caffeine efflux, render budding yeast cells hypersensitive to caffeine, indicating that caffeine tolerance is not solely attributed to its efflux from the cell [96]. In mammals, intracellular caffeine is metabolized and detoxified in the liver by the cytochrome P450 oxidase enzyme system; however, no specific caffeine transporter has been identified [97].

Several caffeine-resistant *S. pombe* mutant strains were isolated in previous studies via mutant screenings [98-100]. Interestingly, all of the identified mutants reacted to caffeine through Pap1, an AP1-like transcription factor in *S. pombe*. Hydrogen peroxide triggers nuclear accumulation of Pap1 for the maintenance of redox homeostasis [101]. Caffeine tolerance was observed in mutants lacking Crm1, the nuclear exporter of Pap1, as well as in mutants defective in Hba1, a cofactor of Crm1-mediated Pap1 export [98, 99, 102]. A loss-of-function mutation in *trr1*, which encodes thioredoxin reductase, induces constitutive oxidation and the constant activation of Pap1 leads to caffeine tolerance. Overexpression of Pap1 itself confers caffeine resistance, but interestingly, caffeine treatment does not enhance Pap1-mediated transcription [98]. These results suggest that Pap1 is responsible for transcriptional regulation of downstream target genes specifically involved in caffeine tolerance or detoxification. In fact, multicopy plasmid-based overproduction of Caf5, which encodes an ABC transporter, causes caffeine resistance and the expression of Caf5 is dependent on Pap1 [103].

Several ABC transporters that mediate multiple drug resistance are known to belong to oxidative stress-responsive regulons. Conversely, many toxic drugs could trigger the production of reactive oxygen species (ROS) in the cell. A genome-wide screening of caffeine-sensitive mutants in *S. pombe* revealed that Pap1 and another H_2_O_2_-induced MAP kinase, Sty1, were both required for caffeine tolerance. Moreover, Pap1-mediated caffeine tolerance is largely due to Hba2, a Pap1-dependent drug efflux pump [50]. However, only the overexpression of Pap1, but not Sty1, can allow a cell to overcome the toxic effects of caffeine, and intriguingly, only the Sty1 pathway, but not Pap1, is activated by caffeine treatment [50, 98]. It is therefore likely that these complex mechanistic changes are not specific to caffeine but might confer protection against a broad range of stressors.

Several caffeine-sensitive mutants are also defective in oxidative stress response mechanisms, and several mutants lacking important components for general stress response, cell wall integrity, vesicle-mediated protein trafficking, and genome stability are both sensitive to H_2_O_2_ and caffeine. This highlights the importance of characterizing the genetic and biochemical crosstalk between the molecular targets of caffeine and oxidative stress signaling pathways to understand the integration of cellular detoxification mechanisms [50]. Overexpression of Yap1, the Pap1 homolog in *S. cerevisiae*, also confers cell resistance to several toxic drugs such as diazaborine and cadmium, and this tolerance is dependent on Ycf1 and Flr1, two multidrug transporters, the expression of which is mainly controlled by Yap1 [104, 105].

## Conclusion

Caffeine is a natural purine analog that has been widely used for studies of cellular response to external toxic materials due to its wide-ranging pleiotropic effects on cells including cell growth, DNA damage repair, and cell cycle regulation, as well as on changes in cell morphology and energy metabolism. Caffeine and other methylxanthine-derived drugs with structural similarities have multiple molecular targets in the cell but possess a particularly high affinity to PI3K-related kinases such as Mec1, Tel1, and Tor1. These inhibitory interactions leading to radiosensitization and cell toxicity presumably require the presence of a specialized group of signaling molecules including PP-IPs and/or properly organized interactions with critical factors involved in homology-directed repair such as Rad51-related nucleoproteins.

Growing evidence suggests that caffeine toxicity could be relieved mainly by several ABC transporters associated with multiple drug resistance, and interestingly, many of them are involved in oxidative stress response as well. Moreover, ROS-responsive transcription factors and a few relevant MAP kinases are genetically- and functionally-linked to caffeine tolerance. In line with these observations, it is strongly suggested that caffeine potentiates cell lethality in conjunction with several other exogenous or endogenous stimuli such as irradiation, toxic chemicals, ROS, and genomic infidelity. Therefore, cells need to be equipped with integrated detoxification mechanisms to maintain physiological homeostasis.

Taken together, the points discussed in this review present a novel framework for future studies to elaborate on the cytotoxic effects of caffeine and its mechanisms of action through its interactions with cell integrity pathways, and could also provide a better understanding of how cells react to caffeine in the context of holistic and pleiotropic stress responses.
